# Biomarkers in Stereotactic Ablative Radiotherapy: Current Evidence and Future Directions

**DOI:** 10.3390/ijms262110640

**Published:** 2025-10-31

**Authors:** Mohamed Metawe, Christos Mikropoulos, Hasan Al-Sattar, Inesh Sood, Amir Mashia Jaafari, Joao R. Galante, Sola Adeleke

**Affiliations:** 1 Royal Surrey Hospital NHS Foundation Trust, Guildford GU2 7XX, UK; mohamed.metawe@nhs.net; 2 School of Medicine and Biomedical Sciences, University of Oxford, Oxford OX1 3EL, UKamir.jaafari@trinity.ox.ac.uk (A.M.J.); 3 The Royal Marsden NHS Foundation Trust, London SW3 6JJ, UK; 4 School of Biomedical Engineering and Imaging Sciences, King’s College London, London WC2R 2LS, UK

**Keywords:** SABR, biomarkers, ctDNA, PSMA+ extracellular vesicles, radiomics, immunotherapy, oligometastatic cancer, liquid biopsy, translational oncology

## Abstract

Stereotactic ablative radiotherapy (SABR) has revolutionized the management of patients with oligometastatic and selected primary cancers due to its ability to deliver highly conformal, high-dose radiation in few fractions with minimal toxicity. However, the biological heterogeneity among patients treated with SABR results in variable outcomes, emphasizing the need for predictive and prognostic biomarkers to guide patient selection and post-treatment management. This narrative review discusses the current landscape of biomarker development in the context of SABR across tumor types. Key classes include circulating tumor DNA (ctDNA), extracellular vesicles (EVs), radiomic features, and immunological markers. We highlight the role of each biomarker category in refining therapeutic approaches, their integration into ongoing clinical trials, and future directions for personalized SABR paradigms. Translating these promising biomarker strategies into clinical SABR workflows will require further standardisation, validation, and regulatory alignment.

## 1. Introduction

Stereotactic ablative radiotherapy (SABR), also known as stereotactic body radiation therapy (SBRT), is now widely accepted as a potentially curative treatment for patients with primary and oligometastatic cancers—a clinical state characterized by limited metastatic burden, often defined as one to five lesions [1]. In addition, the precise delivery of high-dose radiation using SABR achieves excellent local control rates in a range of tumors [2]. However, not all patients derive the same benefit from SABR, and patterns of failure often reveal early systemic progression despite effective local therapy [3].

Such interpatient variability underscores the need for robust biomarkers to predict which patients are most likely to benefit from SABR and to personalize treatment strategies accordingly. Biomarkers can provide critical insights into tumor aggressiveness, microenvironmental features, immunological landscape, and treatment response. Furthermore, as SABR becomes increasingly integrated with systemic agents such as immunotherapy and targeted therapies, biomarkers may help guide treatment sequencing and combination strategies.

## 2. Circulating Tumor DNA (ctDNA)

Circulating tumor DNA has gained attention as a minimally invasive tool for tumor genotyping, disease monitoring, and early detection of relapse.

ctDNA consists of short DNA fragments shed from tumor cells into the bloodstream, primarily through apoptosis, necrosis, and potentially active secretion [4]. During apoptosis, endonuclease-mediated cleavage produces fragments of approximately 160–180 base pairs—corresponding to the length of DNA wrapped around a nucleosome—while necrotic release can generate longer, more heterogeneous fragments [5]. Once in circulation, ctDNA is rapidly cleared via hepatic and renal pathways, giving it a short half-life of around 1–2 h [6]. The fraction of ctDNA within the total pool of cell-free DNA is typically low (often below 1%), which poses analytical challenges requiring highly sensitive detection methods such as digital droplet PCR or next-generation sequencing with error suppression [4]. These assays enable the detection of tumor-specific alterations, including single-nucleotide variants, copy number changes, and methylation or fragmentomic patterns, thereby allowing for ctDNA to serve as a dynamic reflection of the tumor’s genomic landscape and biological activity [7].

Given that these biological characteristics and rapid turnover, ctDNA provides a real-time snapshot of tumor dynamics and can be leveraged in several clinical contexts. Longitudinal plasma monitoring allows for assessment of minimal residual disease (MRD), clonal evolution, and therapeutic response [8]. In the setting of SABR, several studies have investigated the role of ctDNA as a dynamic biomarker.

In patients with oligometastatic renal cell carcinoma, longitudinal monitoring of circulating tumor DNA (ctDNA) has demonstrated potential as a prognostic biomarker, identifying individuals at higher risk of early relapse following SABR [9]. In a prospective registry study, ctDNA was assessed using a tumor-informed assay (Signatera^TM^, Natera, 13011 McCallen Pass, Building A Suite 100, Austin, TX, USA) during post-SABR surveillance. The presence of detectable ctDNA was associated with subsequent radiographic progression, with a sensitivity of 64.7%, specificity of 100%, positive predictive value (PPV) of 100%, and negative predictive value (NPV) of 80.6%. Patients with undetectable or declining ctDNA levels post-SABR were more likely to remain radiographically disease-free, supporting the integration of ctDNA kinetics into post-treatment surveillance to identify candidates for early systemic intervention.

In addition to post-treatment surveillance, ctDNA has also shown promise in refining patient selection for locally consolidative therapy. A large multi-institutional cohort study of 1487 patients with oligometastatic non-small cell lung cancer (NSCLC) evaluated the prognostic value of ctDNA prior to radiotherapy [10]. Among the 309 patients with ctDNA measurements available before treatment, those with undetectable ctDNA (defined as the absence of pathogenic or likely pathogenic variants using a commercial assay) experienced significantly improved progression-free survival and overall survival. Additionally, higher pre-treatment ctDNA variant allele frequency and ctDNA mutational burden were both inversely associated with PFS and OS. These findings suggest that ctDNA can serve as a non-invasive biomarker to distinguish between patients with true oligometastatic disease, who are more likely to benefit from SABR, and those with undetected micrometastatic disease, for whom systemic therapy may be more appropriate.

The SABR-SYNC trial is evaluating the prognostic and predictive utility of ctDNA in patients with synchronous oligometastatic malignancies undergoing SABR [11]. As described in the study protocol, serial peripheral blood samples will be collected to assess cell-free DNA and tumor-derived ctDNA as part of the trial’s translational biomarker program. This approach is intended to explore the role of ctDNA as a non-invasive biomarker for tumor burden, treatment response, and early detection of disease progression in this patient population.

While ctDNA is a promising biomarker for disease monitoring, treatment response and relapse detection, ctDNA assays vary widely in terms of their sensitivity and specificity, which is dependent on numerous factors, including the sequencing platform, sequence depth, DNA extraction efficiency and variant allele frequency (VAF) [12,13].

In terms of sequencing modality, a clear difference is apparent between PCR-based and NGS-based approaches, where the former only allows for the testing of a small number of known genomic alterations at a time, while the latter enables the screening of large numbers of either known or unknown mutations in ctDNA. For example, a study demonstrating a rapid increase in ctDNA levels after a single fraction of fractionated radiotherapy in 3/12 patients with NSCLC hypothesised that this could represent initial destruction of tumour cells, and questioned whether ctDNA levels could be used as a surrogate for early treatment response [14]. They used mutation specific digital PCR assays (targeting KRAS, EGFR and TP53) and defined detectable ctDNA levels as greater than 1 copy of mutant DNA detected, with a significant increase defined as a 30% rise in ctDNA levels between baseline and 24 h post treatment samples. This mutation-specific approach means that unselected ctDNA mutations could be missed and may explain why the rise is only seen in 25% of patients.

Alternatively, a study comparing the performance of four NGS gene panel assays for detecting ctDNA mutations found that the majority of false positive and false negative calls were found below 1% VAF, suggesting that variants in this range should be approached with caution [15]. However, 50% of true positive variants had a VAF of less than 1%, highlighting the need for improved assay detection below this threshold. Such limitations can complicate the establishment of clinically meaningful cut-offs, limiting the utility of ctDNA as a concrete biomarker. Currently, the SABR-DETECT study is underway, which is a prospective cohort study examining whether liquid biopsies measuring ctDNA can predict recurrence in patients undergoing SABR for tissue confirmed and tissue unconfirmed lung tumours [16]. Simultaneously, they will investigate whether ctDNA positivity can be used to aid confirmation of malignancy, using the unconfirmed cohort who have not had tissue-diagnosis of NSCLC. It remains to be seen whether this ctDNA-based liquid biopsy platform will be effective as a biomarker for diagnosing recurrences and/or confirming malignancy in patients without tissue-confirmed disease.

## 3. Extracellular Vesicles (EVs)

Extracellular vesicles, particularly exosomes, are membrane-bound nanostructures released by a variety of cell types, including tumor cells. They mediate intercellular communication by transporting bioactive cargo such as proteins, lipids, and nucleic acids. Owing to their stability in biological fluids and their ability to reflect the molecular characteristics of the originating tumor, EVs have gained interest as non-invasive biomarkers in patients undergoing stereotactic ablative radiotherapy [17].

One of the most clinically relevant applications of EVs in the SABR setting is in oligometastatic prostate cancer. A prospective translational study by Andrews et al. examined plasma levels of prostate-specific membrane antigen-positive extracellular vesicles (PSMA^+^ EVs) in patients with hormone-sensitive oligorecurrent prostate cancer enrolled in the ORIOLE and STOMP-like cohorts [18]. Stratification of patients by baseline PSMA^+^ EV levels revealed that lower concentrations were significantly associated with superior post-treatment outcomes. Specifically, the median biochemical progression-free survival (bPFS) was 26.1 months in the low PSMA^+^ EV group versus 15.0 months in the high group (*p* = 0.005), while the median radiographic progression-free survival (rPFS) was 36.0 versus 25.0 months, respectively (*p* = 0.003). Multivariate analysis confirmed PSMA^+^ EV levels as independent predictors of both bPFS and rPFS after adjusting for PSA, number of lesions, and lesion location. Importantly, combining PSMA^+^ EV levels with baseline PSA further refined prognostic stratification. Patients with concurrently low PSA and low PSMA^+^ EV levels (representing approximately 15% of the cohort) demonstrated the most favourable outcomes, with median bPFS and rPFS not reached during follow-up. This subgroup experienced a significantly reduced risk of biochemical (HR 0.34, *p* = 0.0002) and radiographic progression (HR 0.22, *p* = 0.0001). The predictive value of PSMA^+^ EVs for treatment response was also assessed within the ORIOLE trial. Among patients with low baseline PSMA^+^ EV levels, those who received SABR had a markedly longer bPFS compared to those in the observation arm (24.3 vs. 5.8 months, *p* = 0.003), with a significantly reduced risk of biochemical progression (HR 0.19, *p* = 0.004). Conversely, no benefit from SABR was observed in patients with high baseline PSMA^+^ EV levels, suggesting that PSMA^+^ EVs may help identify individuals most likely to benefit from metastasis-directed therapy.

Compared to ctDNA, extracellular vesicles boast the advantage of being more easily detectable across a wider spectrum of disease stages. However, there is still a lack of universal guidelines surrounding the isolation and characterisation of extracellular vesicles, thus hampering cross-study comparisons and limiting clinical reproducibility [19]. Furthermore, Andrew’s et al. found significantly different levels of tumour-related EVs across two similar patient cohorts which were measured using the same techniques, suggesting differences in storage and handling can further impact reproducibility [18].

## 4. Imaging Biomarkers and Radiomics

Radiomics is an emerging field defined by the extraction of large amounts of quantitative features from standard clinical imaging modalities, including computed tomography (CT), magnetic resonance imaging (MRI), and positron emission tomography (PET), to characterise tumour phenotype, heterogeneity, and treatment response non-invasively. Within the context of SABR, radiomic biomarkers have already shown the potential to predict prognosis, refine patient selection, and facilitate individualised treatment planning.

For example, Cao et al. [20] conducted a retrospective, multi-institutional study involving 117 patients diagnosed with oligometastatic castration-sensitive prostate cancer (omCSPC) and treated with SABR. Radiomic features were extracted from pre- and post-treatment PSMA PET/CT scans, specifically targeting both the gross tumour volume and a peritumoural expansion zone. Using machine learning algorithms, including Random Forest and Support Vector Machine, six radiomic features (e.g., entropy, total energy, standard deviation) combined with five clinical parameters (e.g., PSA, Gleason score, lesion count) achieved an 80% predictive accuracy for 2-year metastasis-free survival (MFS), with an area under the curve (AUC) of 0.82. In cross-institutional validation, the model showed strong discriminatory performance (AUC 0.77–0.80), supporting its application in SABR decision-making. Similar findings were reported by Ong et al. [21], who examined outcomes in a single-centre cohort of 20 men with PSMA PET/CT-confirmed oligometastatic prostate cancer and subsequently treated with SABR. The study demonstrated a 12-month local progression-free survival (LPFS) rate of 93%, an androgen deprivation therapy-free survival (ADTFS) of 70%, and PSA reductions observed in 60% of patients. Although radiomic analyses were not performed, the study highlights the diagnostic value of PSMA PET/CT in lesion delineation and the potential for future radiomics integration.

Beyond prostate cancer, the potential of radiomics to predict prognosis has been documented in other tumour types. For instance, Lubner et al. [22] assessed CT texture features in 77 patients with colorectal cancer liver metastases. Entropy, mean positive pixels (MPP), and standard deviation (SD) were significantly associated with tumour grade and overall survival. Notably, coarse-texture entropy correlated with improved survival outcomes (hazard ratio [HR] for death 0.65; 95% CI: 0.44–0.95; *p* = 0.03), highlighting imaging heterogeneity as a marker of tumour aggressiveness. Similarly, Parmar et al. [23] evaluated prognostic radiomic classifiers in head and neck cancer across two cohorts (*n =* 101 and *n* = 95). Of the 440 extracted features, the most stable and predictive classifiers, including mutual information and minimum redundancy-maximum relevance, achieved AUCs of up to 0.69 for overall survival.

However, the clinical implementation of radiomics is currently limited by methodological inconsistencies and a lack of standardisation. To overcome this, the Image Biomarker Standardisation Initiative (IBSI) evaluated 174 core radiomic features across 25 research teams using phantom datasets and clinical imaging [24]. They established reference values and consensus definitions to enhance reproducibility and allow for standardisation across institutions, representing a significant milestone for radiomics research. Similar efforts by consortia such as the Quantitative Imaging Biomarkers Alliance (QIBA) also focus on addressing limitations by establishing imaging protocols, test–retest reproducibility benchmarks, and performance metrics tailored to cancer applications [25].

Artificial intelligence (AI) could also further improve the radiomic framework by enabling high-throughput feature selection, dimensionality reduction, and model optimisation. Radiogenomics—a computational discipline that integrates radiomic and genomic data—has shown promise in stratifying patients and guiding SABR regimens. Shui et al. [26] explain how radiogenomic signatures may serve as surrogates for molecular tumour characteristics, predicting immune responsiveness as well as resistance mechanisms. Looking ahead, radiomics could also be utilised with other biomarker platforms. As described by Vučinić et al. [27], integrating radiomic features with genomic and immunological data, for example, markers of DNA damage repair pathways, can create models capable of predicting SABR sensitivity, resistance, and optimal combination strategies.

## 5. Immune Biomarkers and Liquid Biopsies

The tumour immune microenvironment plays a critical role in mediating the therapeutic effects of SABR, which is increasingly recognised not only as a local cytotoxic modality, but also as a systemic immunomodulatory intervention [28]. Ionising radiation has already been shown to enhance antigen release, promote dendritic cell maturation, and facilitate the recruitment and activation of cytotoxic T cells, thereby rendering tumours more immunogenic and potentially responsive to immunotherapy [29]. These immunological dynamics form the rationale for combining SABR with immune checkpoint inhibitors (ICIs) and incorporating immune biomarkers into clinical studies to predict response and disease trajectory.

Recent translational research has demonstrated the value of peripheral immune profiling as a biomarker platform. In a prospective observational study by Zafra et al. [30], 27 patients with oligoprogressive non-small cell lung cancer (NSCLC) or melanoma receiving ICI-SABR were assessed using serial blood sampling for cell-free DNA (cfDNA), immune phenotyping of peripheral blood mononuclear cells (PBMCs), and small RNA expression from extracellular vesicles (EVs). Blood was collected at multiple timepoints: baseline, post-SABR fractions, two months post-treatment, and at progression. Responders, defined by immune RECIST criteria, demonstrated a decline in cfDNA from the second to third treatment fractions and a concurrent increase in CD8^+^PD-L1^+^ T cells, while non-responders showed elevated CD8^+^PD-1^+^ T cells (n = 27, response rate 63%, including 26% complete responses). Furthermore, 27 differentially expressed microRNAs were identified post-treatment, offering promise as early predictors of response.

Immune cell subsets in circulation have also been implicated in therapeutic outcomes. Huang et al. [31] conducted mechanistic immune profiling of patients with metastatic melanoma treated with pembrolizumab. Using high-dimensional flow cytometry, they observed that the proliferation marker Ki-67 was significantly upregulated in PD-1^+^ CD8^+^ T cells three weeks after therapy initiation (*p* < 0.0001), signifying T-cell reinvigoration. Notably, the ratio of T-cell invigoration to pre-treatment tumour burden was shown to strongly correlate with clinical response (*p* < 0.01), highlighting its potential as a pharmacodynamic biomarker in checkpoint blockade therapy. This approach may be particularly relevant in the context of ICI-SABR, where radiation-induced neoantigen exposure could amplify T-cell priming.

The capacity of SABR to elicit systemic immune responses, including the abscopal effect, is underpinned by mechanisms of immunogenic cell death (ICD). Demaria et al. [32] described how radiation-induced tumour cell death activates dendritic cells via release of danger-associated molecular patterns (DAMPs), such as calreticulin, HMGB1, and ATP, thereby initiating CD8^+^ T-cell priming. Additionally, SABR treatment has been shown to upregulate chemokines (e.g., CXCL10, CXCL16) and remodel tumour vasculature, resulting in T-cell infiltration. However, these beneficial effects are often counterbalanced by radiation-induced upregulation of immunosuppressive pathways, including the activation of TGF-β and the recruitment of myeloid-derived suppressor cells (MDSCs).

Liquid biopsy technologies further improve biomarker detection by enabling non-invasive and longitudinal monitoring of immune dynamics [33]. Circulating microRNAs (miRNAs), particularly those packaged within EVs, are increasingly proving to be indicators of immune responses and radioresistance [34]. For example, miR-21, miR-155, and miR-146a have been implicated in inflammatory signalling, T-cell function, and modulation of immune checkpoints. Their dysregulation has been associated with tumour progression and resistance to radiotherapy and immunotherapy. Notably, the expression of these miRNAs can change in response to SABR, as demonstrated by Zafra et al., who found that EV-associated small RNAs were differentially expressed post-treatment, offering novel biomarker candidates for tracking treatment response [30].

Tumour mutational burden (TMB), neoantigen load, and T-cell receptor (TCR) diversity can also potentially be used for SABR biomarkers. SABR has been shown to enhance neoantigen release, which may prime a broader and more effective anti-tumour immune response. In the SPARC trial (NCT03603002), Voong et al. investigated changes in TCR clonality and neoantigen-specific T-cell responses in patients with stage I NSCLC receiving SABR [35]. Paired pre- and post-SABR biopsies demonstrated increased infiltration of CD8^+^ T cells and expansion of tumour-reactive clones, suggesting a SABR-induced priming effect at the immune repertoire level.

Despite these advances, the immunological effects of SABR are heterogeneous, and the interpretation of dynamic biomarkers remains a complex process. For example, elevated pro-inflammatory cytokines post-SABR treatment may reflect beneficial immune activation, or this could be classified as immune-related adverse events (irAEs) [36]. Postow et al. [37] describe that irAEs affect diverse organ systems, including the gut, skin, endocrine organs, and lungs, due to systemic immune activation by ICIs. SABR could therefore worsen these adverse effects, highlighting the need for predictive immune monitoring tools.

## 6. The Path to Personalised Oncology

The integration of biomarkers into SABR-focused clinical trials marks a shift in radiation oncology, moving from empirical treatment approaches dominated by a one-size-fits-all archetype to a precision medicine framework. Such an evolution reflects a deeper understanding that oligometastatic disease represents a biologically heterogeneous state requiring personalised therapeutic strategies. Indeed, the systematic incorporation of predictive and prognostic biomarkers into clinical trial design has become essential in optimising patient outcomes with a view to advancing our understanding of the oligometastatic phenotype.

### 6.1. Current Landscape of Biomarker-Embedded SABR Trials

The foundation for biomarker-embedded SABR trials was established by the SABR-COMET study, which provided the first randomised evidence that aggressive local therapy improves overall survival in oligometastatic disease [1]. While SABR-COMET was not designed with integrated biomarker endpoints, its success highlighted the critical need for predictive biomarkers to identify which patients stand to benefit most from aggressive local therapy. In the long-term-follow-up, researchers noted 5-year overall survival was 42.3% in the SABR arm compared to just 17.7% in the control arm, further highlighting the therapeutic potential of SABR in these settings.

Since then, several high-impact clinical trials have set a standard that moves beyond exploratory correlative studies toward biomarker-driven treatment allocation and adaptive designs.

The ORIOLE trial, also a phase II study, highlights how biomarker discovery can be integrated within a randomised controlled framework [38]. This trial validated PSMA-positive extracellular vesicles (EVs) as predictive biomarkers for treatment response and incorporated comprehensive biospecimen collection across multiple timepoints, enabling robust analyses that have informed subsequent trials. Similar insights have been derived from the STOMP trial, another randomised phase II study investigating the role of metastasis-directed therapy in oligometastatic prostate cancer [18]. Researchers demonstrated that specific EV phenotypes, including PSMA+ and EpCAM+ EVs could distinguish responders from non-responders and correlate with disease progression.

SABR-COMET10 takes a fundamentally different approach as a pan-cancer biomarker discovery platform [39]. This direct successor to the landmark SABR-COMET study incorporates comprehensive translational endpoints, including ctDNA analysis, tumour immune microenvironment profiling, and radiomic analysis across multiple tumour types. Importantly, SABR-COMET10 aims to identify universal biomarkers of the oligometastatic state, moving past individual tumour types with a view to more fundamental biology. Similarly, the TAORMINA study in breast cancer demonstrate the integration of multimodal biomarker approaches within pragmatic trial designs [40].

### 6.2. Biomarkers and Big Data: The Importance of Multiomic Integration Computational Frameworks

The complexity of oligometastatic disease dictates a need for multifaceted approaches to biomarker discovery. New approaches leveraging the use of artificial intelligence (AI) have emerged as essential tools for the management of big datasets that integrate genomic, proteomic, metabolomic, and imaging data. Cao et al. highlights the power that can be leveraged from AI by combining quantitative imaging features with molecular profiling to predict treatment response in oligometastatic castration-sensitive prostate cancer (omCSPC) [20]. This multi-institutional study between Johns Hopkins Hospital and Baskent University was able to accurately predict 2-year metastatic-free survival (MFS) by integrating PSMA PET radiomic features with various clinical parameters. Importantly, this study was the first to combine pre-and post-radiomic features with clinical parameters in a way that significantly improved model performance. The combined model achieved an area under the curve (AUC) of 0.89 compared to 0.79 AUC achieved by other models using clinical data alone. In doing so, researchers demonstrated that superior predictive performance can be achieved by combining temporally distinct radiomic signatures with clinical metrics in order to enhance treatment response prediction. Such work underscores the growing role of AI in the personalisation of SABR-strategies.

Another important consideration for biomarker trials of the future is data harmonisation. Harmonisation refers to the alignment of data acquisition, processing, and interpretation methods across institutions and platforms in a bid to reduce technical variability and enable reproducible research outcomes. Without proper harmonisation, interinstitutional variability due to site-specific variation can confound biomarker signals and thus limits the generalisability of findings acting as a hurdle to widespread clinical adoption. The Image Biomarker Standardisation Initiative IBSI is one example of how protocols are established for the extraction of radiomic features in a bid to reduce variation [24]. Furthermore, initiatives like the Blood Profiling Atlas in Cancer BloodPAC are working to standardize liquid biopsy methodologies including ctDNA and EV profiling [41].

The growing number of SABR trials incorporating biomarkers signals a strong commitment to personalised care. As technology advances and we begin to unpack the ‘oligometastatic phenotype’, the role of translational research will be pivotal in transforming SABR from a technical innovation to a biologically guided therapeutic strategy.

## 7. Further Challenges and Future Perspectives

### 7.1. Cost and Accessibility

The widespread clinical adoption of advanced biomarker technologies is also limited by their financial and logistical implications. Many biomarker assays, particularly those involving next-generation sequencing, single-cell technologies, or multiplex imaging, are expensive and require specialized equipment and expertise. A survey of 48 European countries conducted by ESMO highlighted that advanced biomolecular techniques such as whole genome sequencing, genomic assays and liquid biopsies were largely inaccessible in most countries, limited mostly to clinical trials and research in high-income countries [42]. The study also concluded that one of the key barriers to the use of advanced genomic techniques compared to single gene technologies is the financial reimbursement of the test, demonstrating that the costs associated with the development of effective biomarker assays poses substantial challenges for integration in resource-constrained healthcare systems.

As such, cost-effectiveness analyses will be a critical element for evaluating the real-world value of biomarker-guided SABR strategies. A review of the cost-effectiveness of NGS as a biomarker testing approach found that across 29 studies, the costs of NGS ranged from USD 250–7700, with targeted panel testing on the cheaper end of the spectrum (average cost USD 2100) and whole genome sequencing on the more expensive end (average cost of USD 3420) [43]. 9 studies considered the cost-effectiveness of NGS-based biomarker strategies in oncology in terms of holistic testing costs, encompassing both direct testing costs and indirect costs such as personnel requirements, additional hospital visits and additional investigations. All 9 studies concluded that NGS (specifically targeted panel testing) reduced overall costs compared to sequential single gene testing. Furthermore, when considering long term patient outcomes as well as treatment and diagnosis costs in determining cost-effectiveness, 3/13 studies concluded that NGS based biomarker methods are cost effective today, while 4/13 argued moderate cost-effectiveness and expected this to increase as the cost of NGS decreases over time. This suggests that adoption of such biomarker technologies may result in more effective utilisation of healthcare resources in the long term.

Fortunately, biomarkers that do not require expensive assays are also being studied. For example, a study reported that an increase in CD8+PD1+ cells after administration of the first fraction of SABR was seen in responders, whereas a rise in CD8+PDL1+ cells was seen in non-responders [30]. Similarly, another study showed that in patients that received SABR, low neutrophil-lymphocyte ratio (NLR), low platelet-lymphocyte ratio (PLR), and high lymphocyte-monocyte ratio (LMR) before starting treatment were associated with improved overall survival [44]. These potential biomarkers only require measurement of peripheral blood cells, which is relatively inexpensive and accessible in standard laboratories and could thus serve to help predict responses across a wide variety of treatment landscapes.

### 7.2. Regulatory and Ethical Considerations

Complex regulatory and ethical challenges are involved in the implementation of biomarker-guided SABR therapy, which must be navigated carefully to ensure patient safety and public trust. A key challenge lies in the fact that predictive biomarkers will likely have the potential to inform treatment escalation or de-escalation by, for example, identifying potential non-responders who could be placed on a more intense treatment regimen, switched to an alternative therapy or counselled for palliation [45]. This raises ethical concerns as an inaccurate or inadequately validated biomarker could lead to inappropriate over or under-treatment. The former may expose patients to unnecessary toxicity whereas the latter risks suboptimal disease control.

Therefore, regulatory agencies such as the U.S. Food and Drug Administration (FDA), the European Medicines Agency (EMA), and the UK’s Medicines and Healthcare products Regulatory Agency (MHRA) employ strict frameworks for the validation of predictive biomarkers [46]. These typically require multiple lines of evidence including analytical validity (test accuracy and reproducibility), clinical validity (ability to predict outcomes or response), and clinical utility (clear benefit in guiding treatment decisions).

In the context of SABR, biomarker candidates have yet to meet these thresholds, often due to reliance on retrospective data, small cohorts, or insufficiently powered studies [47]. Until biomarkers undergo formal qualification through prospective trials, their use in clinical decision-making remains investigational.

### 7.3. Data Integration and Multimodal Analysis

Developing clinically useful biomarkers to help guide SABR therapy will likely require the integration of heterogenous datasets. Each data modality can provide valuable and complementary information about the effectiveness of SABR therapy, yet the differing data structures, scales and dimensions can complicate efforts to synthesise them into a unified predictive network. Sophisticated data harmonisation, pre-processing and feature selection is necessary to align these diverse datasets effectively.

Artificial intelligence (AI) and machine learning (ML) approaches are useful tools for overcoming this complexity and can help facilitate the development of multimodal predictive models [48]. These tools could be employed to develop predictive models that forecast treatment response, toxicity risk or survival in patients receiving SABR treatment. However, a pre-requisite for the development of such models is the availability of large, high quality and well-annotated datasets, which are currently still in limited supply across SABR cohorts.

### 7.4. Future Perspectives

Despite these challenges, biomarker-driven stereotactic ablative radiotherapy presents an exciting avenue for improving outcomes in oligometastatic patients. To reach clinical implementation of such strategies, future efforts must priorities the development of standardised and reproducible biomarker assays, which are validated across diverse patient populations. The development of these systems will allow for more robust and comparable trials investigating the predictive utility of such biomarkers, which in turn will increase the availability of high-quality, transparent, and independently validated data points. This will provide the necessary input for training multimodal analytical models that can integrate complex biological, imaging, and clinical data and yield multiparametric biomarkers. Therefore, the success of biomarker-guided strategies is clearly dependent on a combination interrelated factors.

At the same time, we must address issues of cost and accessibility to ensure maximal benefit from the development of personalised SABR. Furthermore, regulatory frameworks must continue to prioritise patient safety and ethical integrity, while adapting to the unique challenges presented by the evolving field of personalised biomarker-guided and data-driven therapy. Overall, interdisciplinary collaboration, rigorous validation, and technological innovation will be vital for harnessing the true potential of predictive biomarkers for SABR therapy.

## 8. Conclusions

The evolving landscape of stereotactic ablative radiotherapy (SABR) is being fundamentally reshaped by advances in biomarker science. As SABR transitions from a technical to a biologically informed treatment modality, the integration of predictive and prognostic biomarkers—ranging from ctDNA and extracellular vesicles to immune signatures and radiomic features—holds the promise of truly personalized care.

This review highlights the multifaceted roles of biomarkers across the continuum of SABR, from patient selection and risk stratification to treatment adaptation and longitudinal monitoring. Evidence from translational studies and biomarker-embedded clinical trials demonstrates that these tools can improve outcome prediction, identify early recurrence, and guide therapeutic escalation or de-escalation strategies.

Despite the tremendous promise, several challenges remain. These include the need for standardization, cost containment, regulatory endorsement, and equitable access. To overcome these barriers, collaborative efforts involving clinicians, scientists, regulatory bodies, and patients will be essential. The future lies in integrated multi-omic approaches supported by artificial intelligence and large-scale prospective validation.

In conclusion, the successful incorporation of biomarkers into the SABR paradigm will not only optimize treatment efficacy, but also minimize harm, reduce overtreatment, and usher in a new era of biologically precise radiotherapy. As research progresses, the vision of SABR as a patient-tailored, biomarker-guided therapy becomes increasingly attainable. A summary of the key biomarker classes, their mechanisms, tumour contexts, and translational potential is presented in Table 1.

## Figures and Tables

**Table 1:** Summary of predictive and prognostic biomarkers in stereotactic ablative radiotherapy (SABR): mechanisms, disease contexts, study designs, and performance metrics.

Biomarker (Class)	Biological Rationale/Mechanism	Disease Context and SABR Setting	Study Type and Size	Performance	Clinical Implication	Source
**Tumour-informed ctDNA (Signatera™)**	Plasma ctDNA reflects residual disease burden and clonal evolution after ablative therapy.	Oligometastatic renal cell carcinoma; post-SABR surveillance.	Prospective registry (meeting abstract).	Detectable post-SABR ctDNA associated with radiographic progression; sensitivity 64.7%, specificity 100%, PPV 100%, NPV 80.6%.	Early post-SABR ctDNA positivity flags high relapse risk and candidates for early systemic therapy.	Kwon et al., 2024 [9]
**Pre-radiotherapy ctDNA (VAF/mutational burden/detectability)**	Pre-RT ctDNA indicates occult micrometastatic disease beyond conventional imaging.	Oligometastatic NSCLC; before local consolidative RT/SABR.	Multi-institutional cohort; 1487 total; 309 with pre-RT ctDNA.	Undetectable pre-RT ctDNA associated with longer PFS/OS; higher VAF and ctDNA mutational burden inversely associated with PFS/OS.	Helps distinguish true oligometastatic disease likely to benefit from SABR from biologically polymetastatic disease needing systemic therapy.	Semenkovich et al., 2023 [10]
**Serial cfDNA/ctDNA in biomarker-embedded trial (SABR-SYNC)**	ctDNA kinetics as a non-invasive read-out of tumour burden and response.	Pan-cancer oligometastatic with synchronous primary; SABR-SYNC.	Phase III RCT protocol with translational endpoints.	Protocol specifies serial cfDNA/ctDNA collection; exploratory correlations with outcomes pre-planned.	Prospective platform to qualify ctDNA as a decision tool around SABR.	Palma et al., 2024 [11]
**PSMA-positive extracellular vesicles (EVs)**	Tumour-derived PSMA^+^ EVs index occult tumour burden and metastatic potential.	Oligometastatic/oligorecurrent CSPC; SABR (ORIOLE and STOMP-like cohorts).	Correlative analyses within two randomised cohorts.	Low baseline PSMA^+^ EVs stable longer bPFS (26.1 vs. 15.0 mo; *p* = 0.005) and rPFS (36.0 vs. 25.0 mo; *p* = 0.003); predictive of SABR benefit in ORIOLE.	Prognostic and predictive biomarker to select men most likely to derive durable benefit from SABR.	Andrews et al., 2025 [18]
**PSMA PET/CT-directed lesion selection (imaging biomarker)**	PSMA-avid lesions improve target delineation and identify oligometastatic biology.	Oligometastatic prostate cancer; SABR.	Prospective phase II; n = 20.	12-month LPFS 93%; ADT-free survival 70%; PSA decline in 60%.	Decision-enabling imaging biomarker for SABR planning and surveillance.	Ong et al., 2019 [21]
**PSMA-PET radiomics (features ± clinical)**	Textural/heterogeneity features capture tumour phenotype and peri-tumoural biology.	Oligometastatic castration-sensitive prostate cancer (omCSPC).	Retrospective multi-institutional; n = 117; external validation.	Predictive accuracy for 2-yr MFS ≈ 80%; AUC ~ 0.82 internal; external AUC 0.77–0.80.	Candidate prognostic model to risk-stratify men for SABR and tailor surveillance.	Cao et al., 2024 [20]
**CT texture radiomics (entropy, MPP, SD)**	Imaging heterogeneity correlates with tumour grade and survival.	Colorectal cancer liver metastases (contextual to ablative strategies).	Retrospective; n = 77.	Coarse-texture entropy associated with improved OS (HR 0.65; 95% CI 0.44–0.95; *p* = 0.03).	Radiomics can refine prognosis and potentially SABR candidacy/dose-painting strategies.	Lubner et al., 2016 [22]
**Head and neck radiomic classifiers**	Multi-feature CT radiomics non-invasively stratifies prognosis.	Head and neck cancer (methodological anchor for SABR radiomics).	Two cohorts; n = 101 and n = 95.	Moderate discrimination; AUC ~ 0.61–0.67 across algorithms; stability analyses reported.	Feasibility of robust radiomics pipelines applicable to SABR cohorts.	Parmar et al., 2015 [23]
**Peripheral immune profiling during ICI-SABR (cfDNA, PBMC phenotypes, EV-small RNAs)**	SABR+ICI modulates systemic immunity; cfDNA and PBMC phenotypes track response.	Oligoprogressive NSCLC/melanoma on ICI + SABR.	Prospective observational; n = 27; serial blood sampling.	ORR 63% (CR 26%); responders: cfDNA decline and rise in CD8^+^PD-L1^+^; non-responders: rise in CD8^+^PD-1^+^; 27 small RNAs differentially expressed.	Early liquid-biopsy signals may predict benefit and inform adaptation of SABR-ICI.	Zafra et al., 2024 [30]
**T-cell invigoration to tumour-burden ratio**	Pharmacodynamic biomarker of checkpoint response; potentially amplified by SABR antigen release.	Metastatic melanoma on anti-PD-1 (mechanistic anchor for SABR-IO combinations).	Prospective immune-monitoring study.	Ki-67 upregulation in PD-1^+^CD8^+^ at ~3 weeks (*p* < 0.0001); invigoration:burden ratio correlated with response (*p* < 0.01).	Framework to interpret SABR-induced immune priming and monitor responders.	Huang et al., 2017 [31]
**TCR repertoire dynamics/neoantigen-specific expansion**	SABR can expand tumour-reactive clones and increase CD8^+^ infiltration.	Stage I NSCLC treated with SABR.	Translational paired-sample study.	Post-SABR increase in CD8^+^ infiltration and expansion of tumour-reactive TCR clonotypes.	Supports SABR as an immune-priming modality; rationale for biomarker-guided IO combinations.	Voong et al., 2023 [35]
**Inflammatory indices (NLR, PLR, LMR)**	Systemic inflammation reflects host–tumour interaction and radio-resistance.	Early-stage NSCLC undergoing SABR.	Single-centre retrospective; n = 63.	NLR ≤ 2.06, PLR ≤ 199.6, LMR > 4.0 associated with improved OS; PLR and LMR independent prognosticators.	Low-cost, accessible markers that stratify SABR outcomes; hypothesis-generating for prospective validation.	Luo et al., 2018 [44]

## Data Availability

No new datasets were generated or analyzed in this study.
